# Comprehensive genome sequence analysis of *Ralstonia solanacearum* gd-2, a phylotype I sequevar 15 strain collected from a tobacco bacterial phytopathogen

**DOI:** 10.3389/fmicb.2024.1335081

**Published:** 2024-03-14

**Authors:** Zhiliang Xiao, Guangcan Li, Aiguo Yang, Zhengwen Liu, Min Ren, Lirui Cheng, Dan Liu, Caihong Jiang, Liuying Wen, Shengxin Wu, Yazhi Cheng, Wen Yu, Ruimei Geng

**Affiliations:** ^1^The Key Laboratory for Tobacco Gene Resources, Tobacco Research Institute, Chinese Academy of Agricultural Sciences, Qingdao, China; ^2^Qingdao Agricultural University, College of Agriculture, Qingdao, China; ^3^Fujian Institute of Tobacco Agricultural Sciences, Fuzhou, China

**Keywords:** *Ralstonia solanacearum*, type III effectors, whole-genome sequencing analysis, virulence factors, comparative genomic analysis

## Abstract

**Introduction:**

Plant bacterial wilt is an important worldwide disease caused by *Ralstonia solanacearum* which is a complex of species.

**Methods:**

In this study, we identified and sequenced the genome of *R. solanacearum* strain gd-2 isolated from tobacco.

**Results:**

Strain gd-2 was identified as *R. solanacearum* species complex (RSSC) phylotype I sequevar 15 and exhibited strong pathogenicity to tobacco. The genome size of gd-2 was 5.93 Mb, including the chromosomes (3.83 Mb) and the megaplasmid (2.10 Mb). Gene prediction results showed that 3,434 and 1,640 genes were identified in the chromosomes and plasmids, respectively. Comparative genomic analysis showed that gd-2 exhibited high conservation with ten highly similar strain genomes and the differences between gd-2 and other genomes were mainly located at positions GI12-GI14. 72 type III effectors (T3Es) were identified and RipAZ2 was a T3E specific to gd-2 compared with other eight sequenced strain.

**Discussion:**

Our study provides a new basis and evidence for studying the pathogenic mechanism of *R. solanacearum*.

## 1 Introduction

Plant bacterial wilt is an important worldwide disease caused by *Ralstonia solanacearum*, which can be transmitted through soil, irrigation, plants, and seed potatoes (Genin and Denny, 2012). This pathogen has a wide host range and can infect more than 200 plant species belonging to more than 50 families, including monocotyledons and dicotyledons, such as potatoes, tomatoes, eggplants, peanuts, tobacco, bananas, and ginger (Paret et al., 2010; Qian et al., 2012; Yuliar et al., 2015; Sharma, 2021; Huang et al., 2023). Bacterial wilt is widely distributed in tropical, subtropical, and temperate regions (Elphinstone et al., 2005). Peanut wilt generally causes a 10–20% reduction in production, and, production in severe cases was reduced up to 50% or even halted (Chen et al., 2020). Ginger wilt caused by *R. solanacearum* remains the biggest obstacle to ginger production (Mao et al., 2017). In 1880, Bacterial wilt in tobacco was first discovered in Granville, USA, then it was classified as an important disease in tobacco production subsequently because of the enormous potential threat posed to the tobacco industry (Hayward, 2003). And bacterial wilt in tobacco has subsequently spread throughout the world, including in the United States, Indonesia, Japan, South Korea, and Australia (Prokchorchik et al., 2020).

*Ralstonia solanacearum* has complex physiological and biochemical characteristics. It is a gram-negative rod-shaped bacterium with an optimum growth temperature of approximately 32°C and a pH of 6.6. When cultured on TTC medium, *R. solanacearum* generally exhibits a central reddish color surrounded by a milky white irregular shape and exhibits strong fluidity under high light conditions (Kang et al., 2008). There are two internationally recognized traditional taxonomic methods for *R. solanacearum*. One method divides *R. solanacearum* into five physiological variants based on their host range (Buddenhagen et al., 1962). The other method divides *R. solanacearum* into five biochemical variants based on their utilization of carbohydrates (lactose, maltose, cellobiose, mannitol, sorbitol, and xylitol) (He, 1983). According to the diversity of *R. solanacearum* in different hosts and different geographical origins, Fegan and Prior proposed a new evolutionary taxonomic framework based on the analysis of the 16S-23S rDNA gene spacer region sequence *endoglucanase* (*egl*) gene and *hypersensitive response and pathogenicity* (*hrpB*) gene, which reflects the genetic evolution and geographical origins of *R. solanacearum* better. Its evolutionary taxonomic framework includes four different levels of taxonomic units: species, phylotype, sequevar and clone (Fegan and Prior, 2005). These evolutionary types reflect their different geographical origins: Asian (phylotype I), American (phylotype IIA and phylotype IIB), African (phylotype III), and Indonesian (phylotype IV) (Castillo and Greenberg, 2007). Each evolutionary type can be further subdivided into different sequence types (sequevars), and different sequence types may contain different strains with similar pathogenicity or consistent geographical origins. According to the homology of the *egl* gene sequence in the strains, each evolutionary type of strain is divided into multiple different sequence variants; 55 sequence variants have been identified to date (Liu et al., 2017; Greenrod et al., 2023).

The pathogenesis and regulatory process of *R. solanacearum* are very complex. The main virulence factors include the type I, II, III, IV, V, VI secretion system (T1SS, T2SS, T3SS, T4SS, T5SS, T6SS), extracellular polysaccharides (EPSs) and extracellular proteins (EXPs). Among them, EPSs play a crucial role in pathogenicity of bacterial (Kang et al., 2002; Valls et al., 2006; Tsai et al., 2019). *R. solanacearum* can spread through soil, and it enters the plant roots and invades the vascular bundles of the plant and rapidly spreads to the aboveground tissues through the vascular bundle system. The typical symptoms of diseases caused by *R. solanacearum* infection are browning of the xylem, preferential growth of leaves, and plant wilting. After entering the host, *R. solanacearum* secretes more than 30 effector proteins through the type II secretion system (T2SS), including various cell wall-degrading enzymes. The most studied effector proteins are pectinolytic enzymes and cellulose hydrolytic enzymes, which play an important role in the colonization of *R. solanacearum* (Liu et al., 2005; Tsujimoto et al., 2008; Sharma et al., 2020). The T3SS plays an important role in the interaction between *R. solanacearum* and its host (Alfano and Collmer, 2004; Coll and Valls, 2013). All the type III effectors (T3Es) of *R. solanacearum* are located on the large plasmid of the bacterium, known as the *hrp*, which is approximately 23–30 kb. When this region is mutated, the host cannot exhibit a hypersensitive response or cause plant disease (Mukaihara et al., 2010 Ran et al., 2014). The effector proteins of *R. solanacearum* exhibit widespread gene-level transfer and significant intraspecies genetic differentiation. Peeters et al. (2013a,b) sorted the effector proteins of *R. solanacearum* and unified their nomenclature based on their genetic relationships using the general term Rip to name all T3E genes, obtaining 94 Rip genes and 16 candidate T3E genes. Sabbagh et al. (2019) updated the database published by Peeters et al. (2013a,b) and generated a pangenomic library containing 102 T3Es and 16 hypothetical T3Es. The functions of the effector proteins of *R. solanacearum* include interfering with the basic defense response of plants, interfering with host plant metabolic processes, promoting infection, and stimulating host plant immune responses (Tasset et al., 2010; Landry et al., 2020; Cheng et al., 2021; Schachterle and Huang, 2021).

In recent years, the completion of whole-genome sequencing of *R. solanacearum* has laid the foundation for researchers to elucidate the molecular mechanism of disease pathogenesis at the genomic level. The genome of *R. solanacearum* is approximately 5.8 Mb, dominated by two circular replicons. Housekeeping genes and some virulence genes are located on the chromosomes, while important virulence factors, such as T3SS and EPS which determine the pathogenicity of *R. solanacearum*, are located on megaplasmids. Salanoubat et al. (2002) isolated the *R. solanacearum* strain GMI1000 from tomato plants and completed genome sequencing using this strain as a material, which allowed for further research on its pathogenesis mechanism and identification of plant resistance improvement strategies. Currently, the gene data of the strain can be obtained on three database platforms, NCBI,^1^ Ralsto T3E^2^ and *R. solanacearum* sp.,^3^ which play an important role in analyzing the diversity and evolution of the *R. solanacearum* genome, studying the genes affecting host range, and determining the comprehensive regulatory mechanism controlling bacterial virulence (Guidot et al., 2009; Peeters et al., 2013a,b; Tan et al., 2019). Genomic islands (GI) are an important form of horizontal gene transfer (HGT), which contain genes related to various biological functions. The genes carried by GI can often bring selective advantages to bacteria. According to the different genes contained, GI can be generally divided into virulence islands, drug resistance islands, metabolic islands, symbiotic islands (Shrivastava et al., 2010). Then gene islands, secreted proteins are generally considered when identifying virulence factors, which play a key role in enhancing the pathogenic efficacy of pathogens (Stritzler et al., 2018; Choi et al., 2020). The NCBI database has published the complete draft genome of 145 *R. solanacearum*. These Ralstonia strains were mainly isolated from tomato (GMI1000, FJAT-1458), eggplant (EP1, RS-09-161), pepper (RS-10-244, KACC10709), tobacco (CQPS-1, FQY-4), potato (UY031), sesame (SEPPX05) and plantain plants (UW163) (Salanoubat et al., 2002; Ahn et al., 2011; Cai et al., 2015; Asolkar and Ramesh, 2018). The genomic data of Ralstonia isolated from tobacco include Y45 (phylotype I, sequevar 17), FQY-4 (phylotype I, sequevar 17), CQPS-1 (phylotype I, sequevar 17), FJ1003 (phylotype I, sequevar 14), and SL1931 (race 1, biovar 3 strain) (Cao et al., 2013; Liu et al., 2017). Using the genomics of Ralstonia to understand and explore the regulatory mechanism of virulence differentiation and host adaptation will provide an important theoretical basis for targeted prevention and control of Ralstonia.

In this study, we report the isolation of the *R. solanacearum* strain gd-2 from tobacco plants in Fujian Province, China. This strain belongs to phylotype I sequence 15 and exhibits strong pathogenicity to tobacco. We performed whole-genome sequencing and assembly to obtain the genome framework of gd-2. In addition, we performed functional annotation of the gd-2 genome and compared it with other published *R. solanacearum* genome sequences using comparative genomics analysis to explore whether gd-2 has genome segments or genes related to host specificity, providing new evidence for ultimately analyzing the pathogenic specificity of *R. solanacearum* and the prevention and control of bacterial wilt.

## 2 Materials and methods

### 2.1 Strain gd-2 classification and pathogenicity identification

The bacterial strain gd-2 was isolated from Fujian Province, China, and is preserved by the Tobacco Research Institute of the Chinese Academy of Agricultural Sciences. A bacterial genomic DNA extraction kit (TIANGEN, Beijing, China) was used to extract the genomic DNA of the strain. Polymerase chain reaction (PCR) amplification was performed using a composite PCR of the phylotype type of *R. solanacearum*, and the primers are shown in Table 1. The band information was observed through the gel imager, the tested strain was determined to belong to *R. solanacearum* according to whether there were 759/760 bands, and the evolutionary type of the strain was determined by the size of the band.

**Table 1:** Comparison of core type III effectors (T3Es) genes of strain gd-2 with strain CFBP2957, CMR15, CQPS-1, FQY_4, GMI1000, Po82, PSI07, Y45.

T3E_Name	GD-2 Gene ID	CFBP2957	CMR15	CQPS-1	FQY_4	GMI1000	Po82	PSI07	Y45
RipA1	gene1265	absent	absent	absent	100/98	100/97	absent	absent	100/98
RipA2	pA_gene0050	99/78	100/92	100/98	100/99	100/99	96/78	100/77	100/99
RipA3	pA_gene0823	absent	100/89	100/99	100/98	100/98	99/70	99/81	100/99
RipA4	pA_gene0822	100/75	100/90	100/99	100/98	100/99	100/76	100/80	100/99
RipA5	pA_gene1041	98/78	99/93	100/99	100/99	93/99	99/82	99/81	100/99
RipAA	gene2855	68/74	69/76	absent	99/99	absent	67/77	76/79	99/99
RipAB	pA_gene0793	100/72	100/93	100/100	100/100	100/99	100/70	100/75	100/100
RipAC	pA_gene0794	absent	absent	96/100	100/99	100/99	absent	100/73	96/100
RipAD	pA_gene1572	absent	56/79	absent	66/96	53/97	absent	52/71	53/96
RipAE	gene3155	absent	absent	100/98	100/98	100/98	absent	99/76	100/99
RipAF1	pA_gene0847	absent	absent	100/98	100/96	82/97	absent	absent	100/99
RipAI	pA_gene0831	99/81	100/95	100/100	100/99	62/100	92/81	100/84	absent
RipAJ	gene1300	100/70	100/71	absent	100/98	92/99	absent	absent	100/99
RipAK	gene1017	absent	absent	92/99	92/98	84/99	absent	absent	88/99
RipAL	pA_gene0942	82/80	99/83	84/100	98/100	absent	82/80	100/98	94/100
RipAM	gene0169	89/73	100/93	absent	100/100	100/100	89/73	100/83	100/100
RipAN	pA_gene0824	99/70	100/84	100/98	100/98	100/97	98/70	98/73	100/98
RipAO	pA_gene0790	76/74	100/84	100/98	82/100	98/97	72/73	absent	absent
RipAP	pA_gene1238	100/77	100/81	100/99	absent	100/100	100/78	absent	100/99
RipAQ	pA_gene0784	absent	89/77	100/98	100/99	100/98	absent	absent	100/98
RipAR	pA_gene1256	absent	97/74	100/99	100/98	100/99	absent	57/73	100/99
RipAS	pA_gene1370	99/81	100/88	100/99	99/99	100/98	absent	absent	100/99
RipAU	pA_gene1443	82/74	83/80	83/99	83/100	83/99	58/70	absent	100/100
RipAV	pA_gene0934	99/86	100/87	100/97	100/99	100/98	83/86	absent	100/98
RipAW	pA_gene1459	absent	100/84	100/98	100/99	100/95	absent	absent	100/99
RipAX2	pA_gene0569	absent	absent	absent	absent	absent	absent	absent	absent
RipAY	pA_gene1039	absent	98/86	absent	100/97	100/96	absent	absent	100/99
RipAZ1	pA_gene1559	98/81	absent	100/99	100/99	100/99	95/83	97/91	100/99
RipAZ2	gene2616	absent	absent	absent	absent	absent	absent	absent	absent
RipB	gene3236	absent	absent	absent	absent	absent	absent	absent	59/100
RipBD	gene2483	absent	absent	100/100	absent	absent	63/94	absent	84/100
RipC1	pA_gene1259	absent	absent	absent	100/99	100/99	98/70	100/95	100/99
RipC2	pA_gene0585	absent	absent	absent	100/100	100/98	absent	absent	100/100
RipC2	gene2535	92/81	absent	100/83	97/85	97/82	absent	absent	96/75
RipC2	pA_gene0586	absent	absent	absent	100/98	79/99	absent	absent	absent
RipD	pA_gene0241	absent	absent	100/91	100/98	100/98	absent	100/82	100/99
RipE1	gene0066	100/85	96/87	100/99	100/99	100/98	94/85	100/79	94/99
RipE2	gene2507	100/88	absent	absent	absent	absent	100/89	100/98	100/99
RipF1_1	pA_gene1532	100/78	100/94	100/99	100/99	100/99	93/83	100/77	99/99
RipF1_2	pA_gene0754	100/81	100/89	100/81	100/81	100/98	93/76	100/83	100/99
RipG1	pA_gene0742	absent	absent	99/99	100/99	100/99	absent	absent	95/99
RipG2	pA_gene0991	100/72	99/80	100/97	100/98	100/98	100/72	absent	100/97
RipG3	pA_gene0027	absent	86/84	86/99	100/95	86/95	absent	absent	79/95
RipG4	gene1804	absent	100/80	absent	100/98	100/99	absent	absent	100/99
RipG5	gene1805	absent	99/87	100/98	97/98	97/98	absent	97/73	100/99
RipG6	gene2000	99/70	99/85	99/97	99/97	99/78	98/70	98/71	100/97
RipG7	gene1999	absent	absent	absent	absent	absent	absent	99/72	100/99
RipH1	gene1971	absent	99/82	100/97	100/98	100/96	absent	absent	100/99
RipH2	pA_gene0161	absent	98/82	100/97	100/98	100/96	absent	absent	absent
RipH3	pA_gene0104	100/75	100/86	100/95	100/95	100/95	96/78	98/70	100/95
RipI	gene3456	99/85	100/87	100/98	100/98	100/99	99/82	100/93	100/99
RipJ	gene1270	absent	absent	100/94	100/95	69/97	absent	absent	69/97
RipL	pA_gene0139	absent	99/81	100/97	100/97	99/97	absent	absent	96/98
RipM	gene1879	98/72	99/88	absent	100/98	100/99	99/82	99/80	100/98
RipN	pA_gene1159	99/75	100/89	100/98	100/98	100/98	99/74	99/73	100/97
RipO1	pA_gene0263	85/85	100/91	100/100	95/99	88/99	85/86	absent	100/98
RipQ	pA_gene1300	100/80	absent	100/97	100/99	100/99	94/71	absent	100/99
RipR	pA_gene1305	99/83	99/91	absent	100/99	100/99	100/79	100/82	100/99
RipS1	gene0034	96/87	96/90	97/71	100/72	94/99	96/88	96/75	96/99
RipS2	pA_gene1356	97/73	99/91	absent	99/99	93/98	93/74	95/77	97/98
RipS3	pA_gene0726	99/75	99/91	100/99	100/98	96/98	99/75	99/77	100/99
RipS4	gene1840	99/72	96/82	100/99	100/99	98/98	98/71	absent	100/99
RipS5	pA_gene0237	absent	100/85	100/99	100/99	100/99	100/80	99/75	100/99
RipS6	gene1271	absent	absent	absent	100/99	95/98	absent	absent	99/99
RipS8	gene1843	absent	absent	99/98	99/98	absent	absent	98/89	95/98
RipTAL	gene1818	absent	absent	83/98	100/85	83/98	absent	99/74	89/99
RipTPS	pA_gene0935	100/79	100/95	100/99	94/100	100/99	100/78	94/77	100/100
RipU	pA_gene1235	65/73	absent	absent	100/98	absent	65/79	65/78	65/99
RipV1	gene2007	absent	100/83	100/99	100/98	100/99	absent	100/70	100/95
RipW	gene0660	100/76	100/89	100/99	100/99	100/98	100/76	100/81	100/99
RipX	pA_gene0792	absent	100/76	100/95	100/100	100/95	absent	94/77	100/95
RipZ	pA_gene1049	absent	87/83	absent	100/98	100/98	absent	91/71	100/98

Three tobacco cultivars hongda, CHB and K326 were used for pathogenicity test of tobacco bacterial wilt for gd-2, which were common resistance and susceptible control variety (Li et al., 2015 Cao et al., 2013; Pan et al., 2021). And hongda existed high susceptible (HS) to susceptible (S) for bacterial wilt, CHB existed S and K326 existed middle resistance (MR) to resistance (R), respectively. After being activated for 36 h by inoculation and streaking on a TTC culture medium plate, single colonies were picked up with disposable inoculation rings and inoculated into NB liquid medium. The inoculated medium was placed in a shaker at 28°C and 220 r/min for 24 h, yielding a bacterial solution with a concentration of approximately 1.0 × 10^9^ CFU/mL (OD_600_ of 1.0). The bacterial solution was diluted with deionized water to 1.5 × 10^8^ CFU/mL. When the tobacco seedlings reached the five-leaf stage, 30 mL of diluted bacterial suspension was introduced into the bottom of each seedling at a standard rate, keeping the temperature of the bacterial solution at approximately 30°C and the humidity at approximately 80%. Twenty seedlings were inoculated for each variety, and the incidence of bacterial wilt was investigated at 3, 6, 9, 12, 15, and 18 days after inoculation.

### 2.2 Genomic sequencing and assembly

The third-generation sequencing technology platform PacBio Sequel II sequencer (Pacific Biosciences^4^) was used for genome sequencing, which was sequenced by Shanghai Winnerbio Technology Co., Ltd. (Shanghai, China) using Illumina NovaSeq 6000. The sequencing strategy was single-molecule real-time (SMRT) sequencing. Subsequently, the genome sequencing data were analyzed by GC depth analysis and K-mer frequency distribution analysis to determine whether there was contamination from other species or large fragments from other sources. The third-generation PacBio Sequel II platform and the second-generation sequencing platform Illumina NovaSeq 6000 were used to construct large fragment libraries (10–20 kb) and small fragment libraries (~400 bp) from DNA samples that passed quality control. The raw data were obtained by sequencing on different platforms. Canu V2.2^5^ was used to assemble the third-generation data independently. Then, the final assembly result was corrected by using Pilon V1.24^6^ and the second-generation data to obtain the final assembly of the bacterial genome.

### 2.3 Gene prediction and analysis

The assembled genome sequence was used to predict the coding sequence (CDS) of coding genes using Glimmer3^7^ software, transfer RNA (tRNA) genes using tRNAscan-SE^8^ software, and ribosomal RNA (rRNA) genes using Barrnap^9^ software. Gene function annotation was performed by comparing the protein sequence file of the gene with the database. The relevant databases included the Nonredundant Protein Database (NR), Swiss-Prot database,^10^ Pfam database,^11^ Gene Ontology (GO) database,^12^ and Kyoto Encyclopedia of Genes and Genomes (KEGG) database.^13^ DIOMAN^14^ was used for sequence alignment analysis.

Circos Version 0.69-6^15^ software was used to draw genome circles for the obtained chromosomes and plasmids. The default scanning map ordered all scaffolds from large to small or from small to large and concatenated them into a sequence to draw circles without distinguishing direction. The default information from the outer to the inner circle corresponded to genome size identification, positive-and negative-strand gene information, noncoding RNA (ncRNA), GC content, and GC skew. In addition, IslandViewer 4^16^ was used for genome island prediction using IslandPath-DIMOB, Islander, and other methods. Prophage prediction was carried out using PHASTER,^17^ which predicted prophage regions in the chromosome sequence of *R. solanacearum* and analyzed their related genes. CRISPRFinder (Ibtissem et al., 2007) was used to identify all potential CRISPR sequences on the genome, showing their location, the base composition of repeats, and the base composition of spacers. Virulence proteins defined as virulence properties molecules with invasive and toxic properties produced by bacteria, viruses, fungi. They are mainly used to enter host tissue cells by suppressing or evading host immune responses when microorganisms infect hosts and obtain nutrients and self-proliferation from hosts. The Virulence Factors of Pathogenic Bacteria (VFDB) database^18^ was used for virulence gene prediction. The screening thresholds used were a similarity of 80% or greater, a coverage of 60% or greater, and an E value of 1e-5. The comprehensive antibiotic resistance database (CARD^19^) was used for comparative analysis and screening of candidate drug resistance-related genes using screening thresholds of 80% similarity, 60% coverage, and an E value of 1e-5. Based on the Pathogen Host Interactions (PHI-base) database, DIAMOND software (screening threshold E value ≤1e-5) was used to align the amino acid sequence of the sequenced strain with the database. TMHMM (TMHMM Server V 2.0^20^) software was used to predict transmembrane proteins and their structural information among five common transmembrane structures: (a) type I transmembrane, (b) type II transmembrane, (c) multipass transmembrane, (d) lipid chain-anchored membrane, and (e) GPI-anchored membrane. The SignalP^21^ tool was used to predict the signal peptide region of each protein and then combined with the analysis results of transmembrane domains to select proteins with a signal peptide structure but without a transmembrane domain as candidate secreted proteins. The secondary metabolite synthesis gene clusters of the samples were predicted using the antiSMASH bacterial database.

### 2.4 Comparative genomic analysis

We conducted comparative genomic analysis via two methods to further investigate the structural characteristics and key genes of plasmids. One method identified plasmid-specific regions and mutation hotspots through comparative genomic circle diagrams, and the other method identified the structural differences of local gene clusters through gene cluster comparison. We conducted comparative analysis of the 10 reference genome sequences with the large plasmid in the gd-2 genome using BRIGV0.9.5 software^22^ and constructed comparative genomic circle diagrams. In addition, we used EasyFigV2.2.3^23^ to conduct detailed comparative gene cluster analysis of the target region.

### 2.5 Virulence genes analysis

In this study, we focused on analyzing the T3SS, T4SS, T6SS, as these secretion systems and effectors are often closely related to the pathogenic ability of bacteria. The annotation analysis was conducted to annotate T6SS using the software T6SS_finder with thresholds of identity ≥50% and E value ≤1e^−5^. The annotation analysis of the T3Es was conducted using BLAST+ with more than 80% identity and more than 60% gene alignment coverage. Based on the Type VI effector database summarized in the SecReT6 database, annotation analysis of the Type VI effector of the strain gd-2 was conducted using BLAST+ with over 80% identity and over 60% gene alignment coverage. Through HMMER3 software, various genes in the two-component signal transduction system in the genome were obtained and analyzed using the Pfam database combined with the structural domain characteristics of histidine kinases and response regulatory proteins. The genes were divided into three categories, regulator, sensor, and hybrid. We also analyzed the number of chemotaxis genes and recorded their detailed annotation information. The quorum sensing genes were analyzed to identify genes and pathways related to quorum sensing by comparison and analysis with the KEGG database.

### 2.6 Comparative analysis of T3Es in different *Ralstonia solanacearum* strains

The effector protein of the T3SS system in *R. solanacearum* is the main determinant protein of its pathogenicity. We identified the distribution and variation information of T3Es in gd-2 and nine other sequenced and published *R. solanacearum* genomes, including phylotype I strain GMI1000 (BioProject: PRJNA13); phylotype IIA strain CFBP2957 (BioProject: PRJNA224116); phylotype III strain CMR15 (BioProject: PRJEA50681); phylotype IIB strain Po82 (BioProject: PRJEA50683); phylotype IV strain PSI07 (BioProject: PRJNA66837); and three phylotype I *R. solanacearum* sequence variants that can infect tobacco, including sequevar 13 strain CQPS-1 (BioProject: PRJNA331070), sequevar 17 strain FQY_4 (BioProject: PRJNA182081), and sequevar 54 strain Y45 (BioProject: PRJNA224116). The localized T3E database to identify T3Es in nine published *R. solanacearum* genomes were constructed firstly. Using BLAST+, the blast stragegy were the E value 1e-^5^, the over 60% coverage and the over 80%identity. And the common and unique T3Es among the nine *R. solanacearum* strains were compared using the Venn diagram to count the gene distribution and sequence variation of the T3Es in gd-2, the candidate T3E genes of the other eight strains were aligned using BLAST+. We statistically analyzed the functional genes related to the *hrp* gene cluster in all nine strains, extracted the protein sequence files of the related genes and performed comparative display the genomes of the main *R. solanacearum* strains.

## 3 Results

### 3.1 Identification and pathogenicity detection of *Ralstonia solanacearum* gd-2

**Figure 1:**
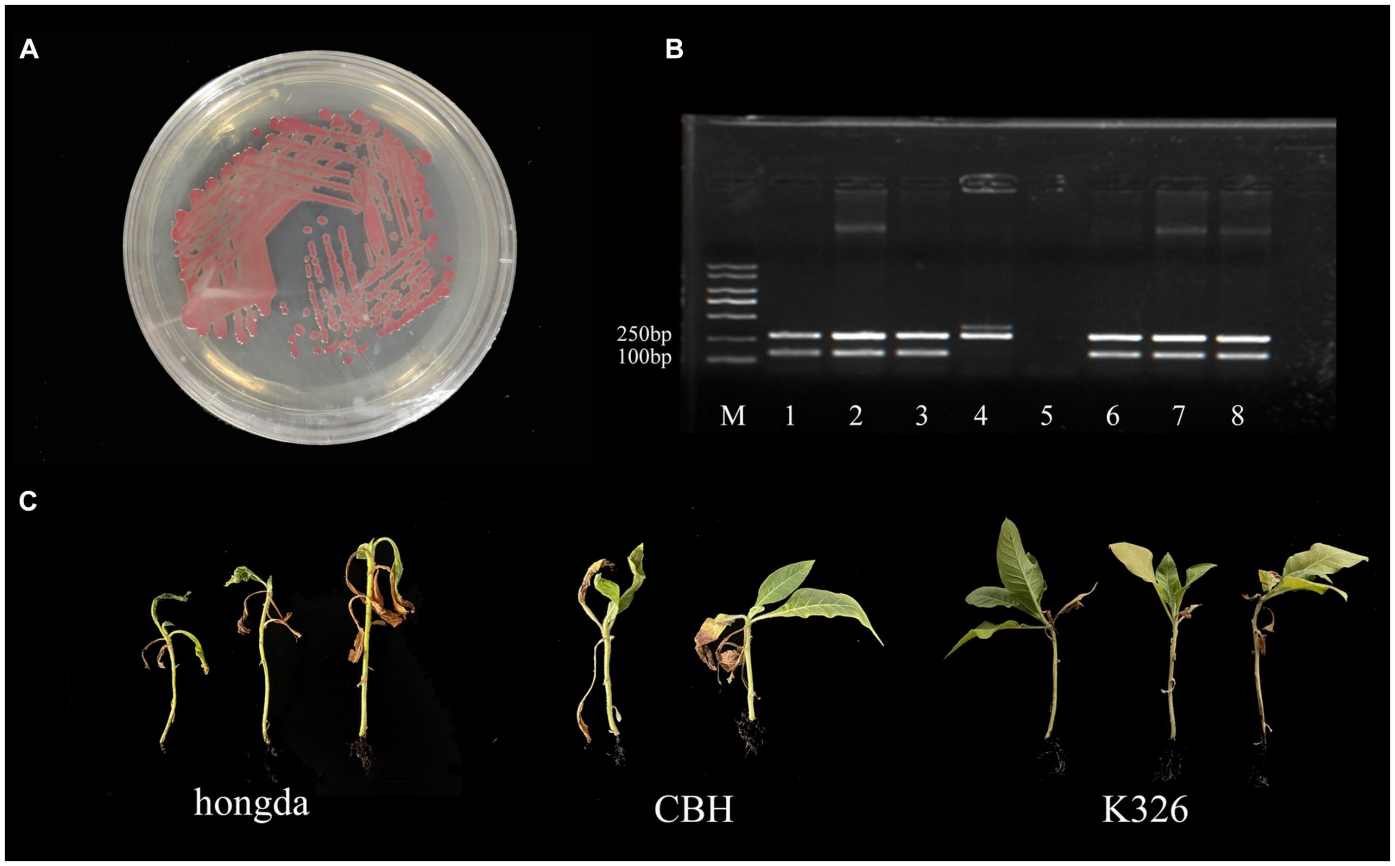
Phenotype, sequence variation, and infectivity identification of the gd-2 strain in tobacco. **(A)** Growth status of the gd-2 strain on TTC plates. **(B)** Electron microscopy observation of gd-2. Line M:marker 5000, line 1: Y45 (phylotype I, sequevar 17), line 2: FQY-4 (phylotype I, sequevar 17), Line 3: gd-2 (phylotype I, sequevar 15), Line 4: AM (phylotype II), Line 5: control, Line 6: HBES (phylotype I, sequevar 44), Line 7: SY-1 (phylotype I, sequevar 17), Line 8: SY-2 (phylotype I, sequevar 15). **(C)** Infectivity of gd-2 on different types of tobacco (hongda, CHB and K326).

The strain gd-2 cultured on TTC medium exhibited a central reddish color surrounded by a milky white irregular shape, and its mobility was visible under high light conditions (Figure 1A). Agarose *gel* electrophoresis showed that the *R. solanacearum* strain gd-2 exhibited 144 bp and 280 bp bands, indicating that the strain is *R. solanacearum* phylotype I (Figure 1B:line 3). Amplification of the *egl* gene of the *R. solanacearum* strain gd-2 using the *endoglucanase*-specific primers Endo-F/Endo-R resulted in an 800 bp band, and sequencing and alignment results showed that the strain belonged to sequence variant 15 (Supplementary Table S2). After inoculating different varieties of tobacco with gd-2, a typical symptom of bacterial wilt was observed: leaves gradually showed wilting symptoms, and the infection spread from the lower leaves to the upper leaves. The stems gradually decayed until the entire tobacco plant died. There were differences in resistance among the tobacco varieties (Figure 1C) and the disease index and resistance performance are similar to other study results of different strains (Li et al., 2015 Cao et al., 2013; Pan et al., 2021). These results indicated that gd-2 meets the characteristics of typical *R. solanacearum* and has pathogenicity to tobacco.

### 3.2 Sequencing, assembly and annotations of the gd-2 genome

**Figure 2:**
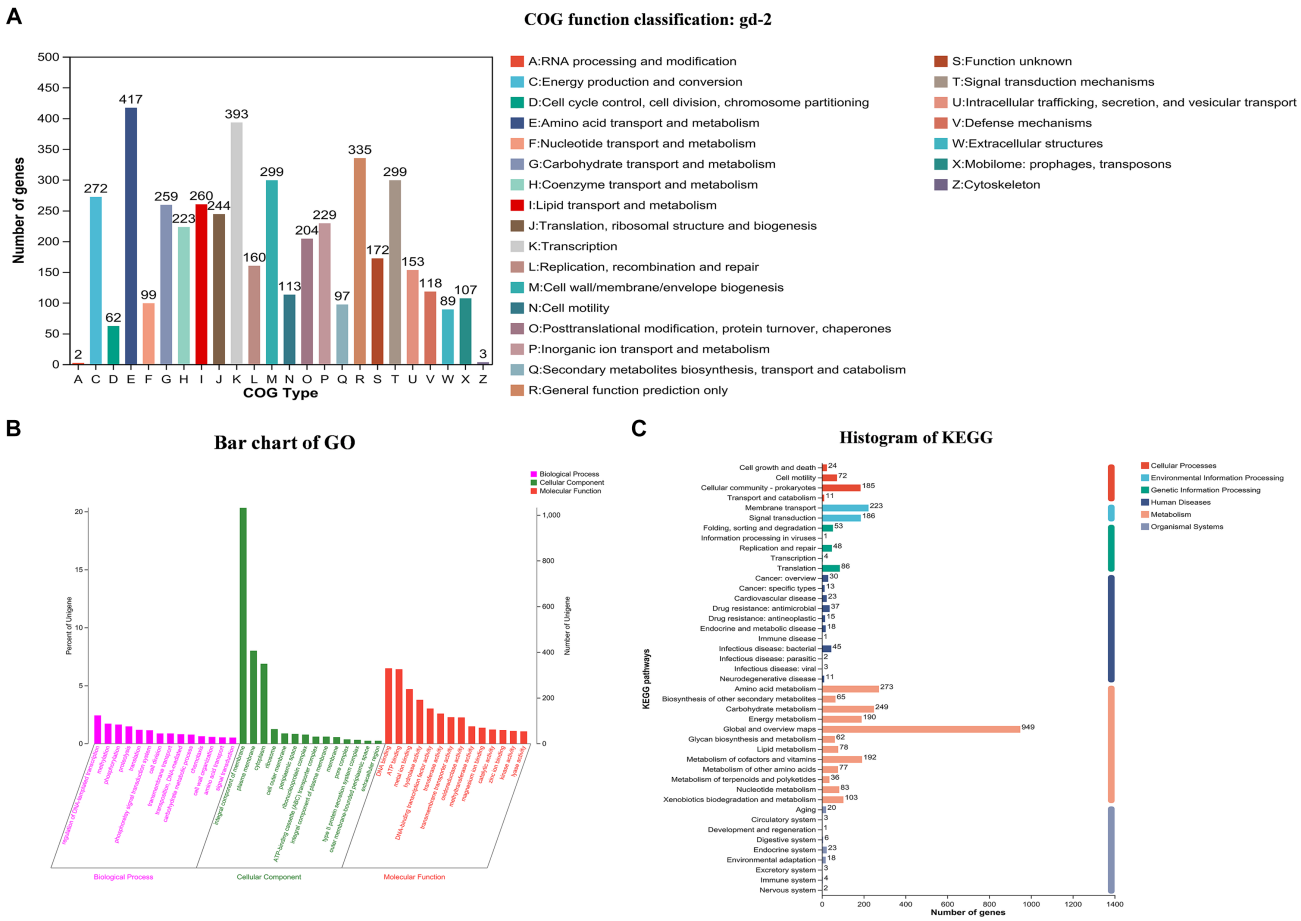
COG, GO and KEGG functional categories of protein-coding genes in the *R. solanacearum* gd-2 genome. **(A)** COG classification of gd-2. The horizontal axis is the 25 COG categories, and the vertical axis is the number of genes annotated to the relevant categories in the genome. **(B)** Clustering results of GO annotation. The horizontal axis is the 3 GO categories, and the vertical axis is the number of genes annotated to the relevant categories in the genome. **(C)** Histogram of KEGG analysis of gd-2. The horizontal axis is the number of genes, and the vertical axis is the number of genes annotated to each category in the genome.

Total 7,666,395,879 bp base reads were identified from sequencing data. The GC depth and K-mer frequency distribution results showed that there was no contamination of miscellaneous bacteria in the sequencing data. After assembling the sequencing data, 3,828,519 bp chromosomes and 2,098,962 bp plasmids were obtained. Gene prediction results showed that 3,434 and 1,640 genes were identified in the chromosomes and plasmids, respectively. The predicted noncoding RNAs included 59 tRNAs, 12 rRNAs, four 5S rRNAs, four 16S rRNAs, and four 23S rRNAs. Different databases identified 2,600–5,000 genes with functional annotations. The COG annotation classification, GO annotation classification, and KEGG annotation classification are shown in Figure 2. COG annotation classification involved 24 categories, and the five categories with the largest number of genes were amino acid transport and metabolism (417), transcription (393), general function prediction only (335), signal transduction mechanisms (299), and cell wall/membrane/envelope biogenesis (299); 172 genes had an unknown function. For GO annotation classification, the three biological process categories with the highest number of enriched genes were regulation of DNA-templated transcription, methylation, and phosphorylation; the three cellular component categories with the highest number of enriched genes were integral component of membrane, plasma membrane, and cytoplasm; and the three molecular function categories with the highest number of enriched genes were DNA binding, ATP binding, and metal ion binding. For KEGG annotation classification, global and overview maps, signal transduction, and membrane transport had the highest number of enriched genes.

**Figure 3:**
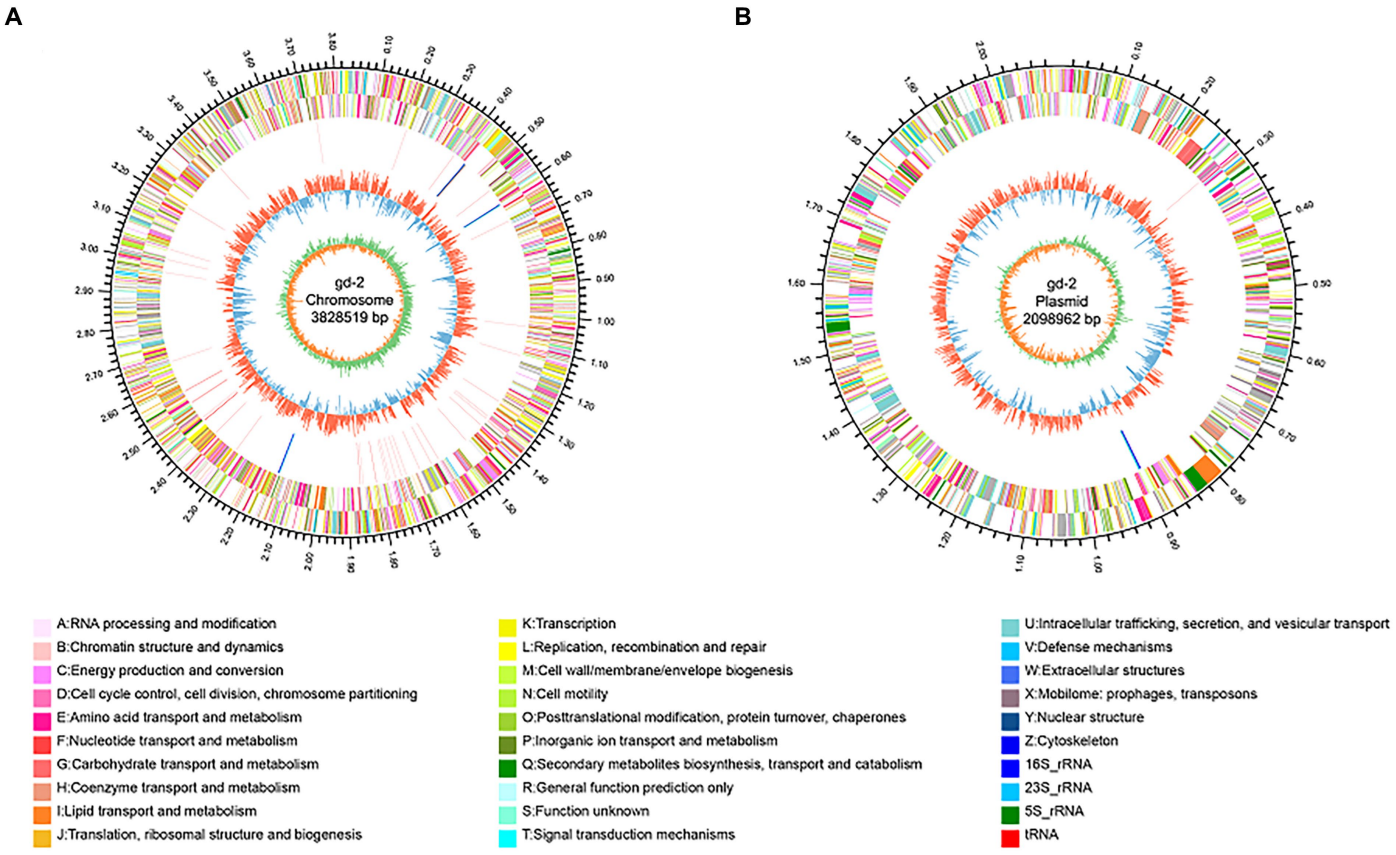
Genomic circle diagram of gd-2 (chromosome and plasmid). **(A)** Genomic circle diagram of gd-2. **(B)** Plasmid circle diagram of gd-2. The outermost circle of the circle diagram indicates the size of the genome; the second and third circles are the CDS on the positive and negative strands, with different colors indicating different functional classifications of COGs for the CDS; the fourth circle is rRNA and tRNA; the fifth circle is GC content, with outwards red portions indicating high GC content in the region. The higher the peak value is, the greater the difference from the average GC content. The inner blue part indicates that the GC content in this region is lower than the average GC content of the whole genome. The higher the peak value is, the greater the difference from the average GC content. The innermost circle is the GC skew value, which is calculated as G-C/G + C and can assist in determining the leading and lagging strands. Generally, the leading strand GC skew >0 and the lagging strand GC skew <0. It can also assist in determining the replication start point (minimum cumulative deviation) and end point (maximum cumulative deviation).

The genome circle diagram include genome size identification, gene information on the positive and negative strands, ncRNA, GC content, GC skew, and other information (Figure 3). The genes carried by GIs usually confer selective advantages to bacteria. 17 GIs were identified, of which 11 originated from chromosomes and 6 from plasmids (Supplementary Table S3). Seven prophages were identified and of which six originated from chromosomes and one from plasmids (Supplementary Table S4). Three CRISPR_Cas systems were identified, of which one originated from chromosomes and two from plasmids (Supplementary Table S5). In addition, 130 genes were annotated as carbohydrate active enzyme-related genes (Supplementary Table S6), 68 genes were predicted as pathogen–host interaction-related genes (Supplementary Table S7), 1,172 genes were predicted to have transmembrane structures (Supplementary Table S8), 494 genes were predicted to be transporters (Supplementary Table S9), 848 genes contained signal peptide domains (Supplementary Table S10), and 705 genes were predicted to be secreted proteins (Supplementary Table S11).

### 3.3 Comparative genome analysis between the strain gd-2 and 10 highest similarity genomes

**Figure 4:**
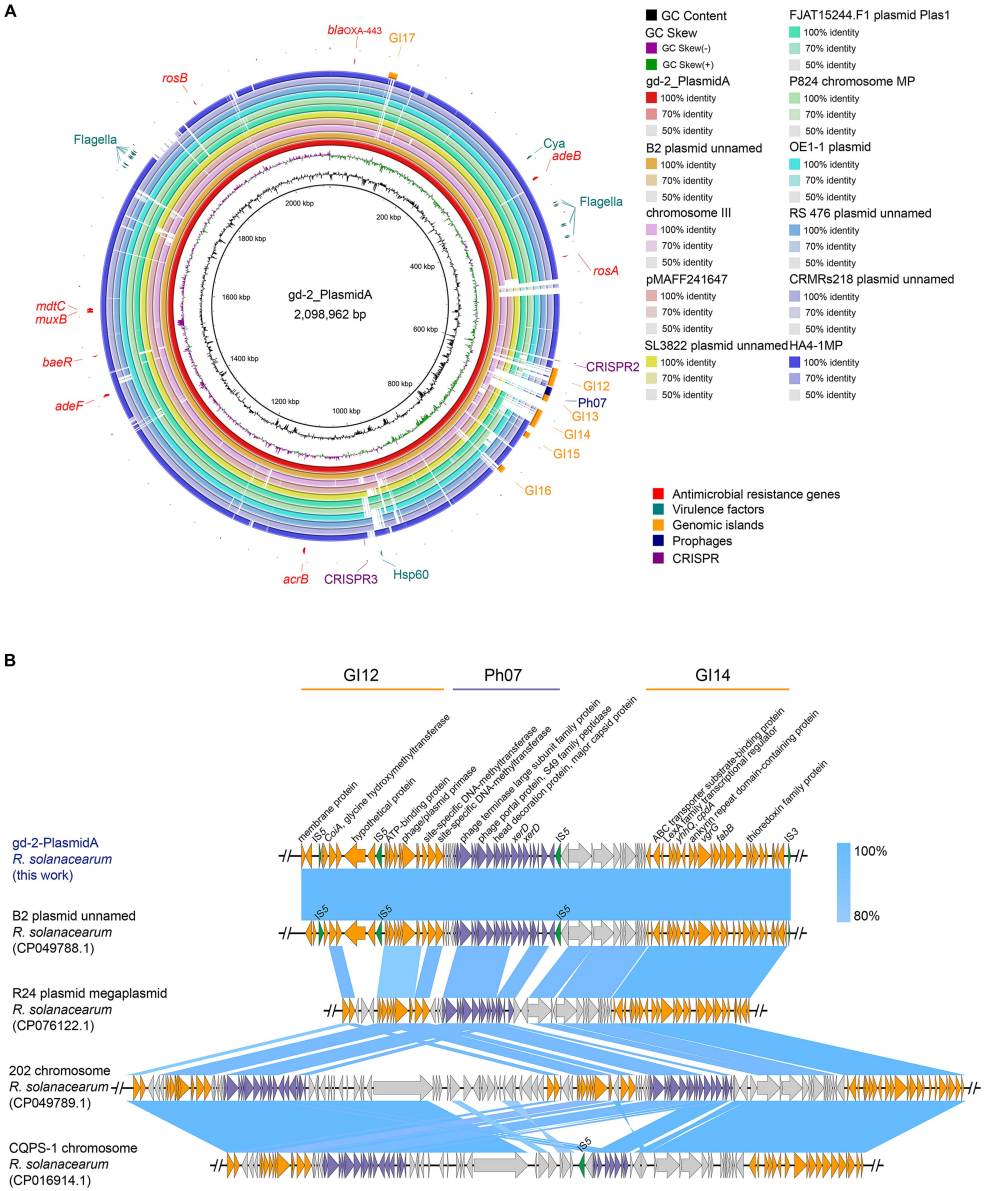
Comparison of genome circle analysis and plasmid comparative gene cluster analysis of gd-2. **(A)** Comparison of genome circle analysis of gd-2. (1) The large plasmid in the gd-2 genome is used as the reference genome, and the order from inside to outside is as follows: first circle: the GC content of the gd-2 plasmid; second circle: the GC skew of the gd-2 plasmid; third circle: the full-length sequence of the gd-2 plasmid; fourth circle: the full-length sequence of the B2 genome plasmid; fifth circle: the full-length sequence of the Phyl III-seqv23 genome chromosome III; sixth circle: the full-length sequence of the pMAFF241647 plasmid; seventh circle: the full-length sequence of the SL3822 genome plasmid; eighth circle: the full-length sequence of the FJAT15244.F50 genome Plas1 plasmid; ninth circle: the full-length sequence of the MP chromosome in the P824 genome; tenth circle: the full-length sequence of the plasmid in the OE1-1 genome; eleventh circle: the full-length sequence of the plasmid in the RS 476 genome; twelfth circle: the full-length sequence of the HA4-1MP plasmid; thirteenth circle: the mobile elements annotated on the gd-2 plasmid, including genomic islands, prophages, and CRISPR–Cas systems; fourteenth circle: the suspected drug resistance genes annotated on the gd-2 plasmid; and fifteenth circle: the suspected virulence genes annotated on the gd-2 plasmid. (2) Comparison of plasmid comparative gene cluster analysis of gd-2-PlasmidA. Sequence comparison analysis of GI12-GI14 of gd-2-PlasmidA with B2, 202, CQPS-1.

By BLAST alignment of gd-2 genome data with the NCBI database, 10 sequences with the highest similarity were identified, including six plasmid sequences of *R. solanacearum*, two chromosome sequences of *R. solanacearum*, and two plasmid sequences of *R. pseudosolanacearum* (Supplementary Table S12), the comparative genome circle diagram was shown in Figure 4A. The full length of gd-2-PlasmidA is 2,098,962 bp, with a GC content of 67.00%. Screening genes with identity >60% and coverage >90% predicted by CARD and VFDB, we identified nine possible antibiotic resistance genes and 18 candidated virulence factors. The antibiotic resistance genes are mainly related to various multidrug efflux pumps, such as *adeABC* gene *adeB*, *RosAB* gene *rosA* and *rosB*, *AcrAB-TolC* gene *acrB*, *AdeFGH* gene *adeF*, *MuxABC-OpmB* gene *MuxB*, *MdtABC-TolC* gene *MdtC* and *BaeR* which promotes the expression of *MdtABC* and *AcrD* efflux pumps. The virulence facors include *flagella* which encoding polar flagella needed for motility and macrophage invasion, *Cya* which encoding a dual-function toxin with adenylate cyclase and haemolytic activity, contribute as an anti-inflammatory protein and heat shock protein (Hsp) 60 which mediates complement-independent attachment to mammalian and amoebic host cells. In addition, five GIs, one prophage and two CRISPR elements were also identified on this plasmid. The differences between gd-2-PlasmidA and other genomes were mainly located at the position of GI12-GI14 (612,748–700,708 bp).

We performed a detailed comparative gene cluster analysis of the GI12-GI14 region of gd-2-PlasmidA with four highest similar strain B2 plasmid (GenBank: CP049788.1), strain R24 plasmid megaplasmid (GenBank: CP076122.1), strain 202 chromosome (GenBank: CP049789.1), and strain CQPS-1 chromosome (Figure 4B). The analysis showed that the GI12 sequence of gd-2-PlasmidA showed some differences in the two putative proteins before the second IS5 and parts of IS5 from other sequences except for the B2 plasmid. In contrast, the GI14 sequence showed high similarity among the various strains, although the six genes at the end of the chromosome were deleted in both strain 202 and strain CQPS-1. In addition, the prophage structure of the R24 plasmid megaplasmid showed the greatest difference from strain Ph07 of gd-2-PlasmidA, with the internal deletion of three consecutive *XerD* genes, which were inverted in the chromosome sequences of strain 202 and strain CQPS-1. Chromosome of strain 202 contained two copies of the same Ph07 strain-like sequence while chromosome strain CQPS-1 contained an additional copy of the Ph07 strain-like sequence, but it lacked many other genes. These results reflect that the mobile elements of GI12, Ph07 and GI14 may have undergone further gene loss or multiple gene copies after integration into the gd-2-PlasmidA-like plasmid of *R. solanacearum* resulting in significant differences in the plasmid.

### 3.4 Virulence genes of the strain gd-2

**Figure 5:**
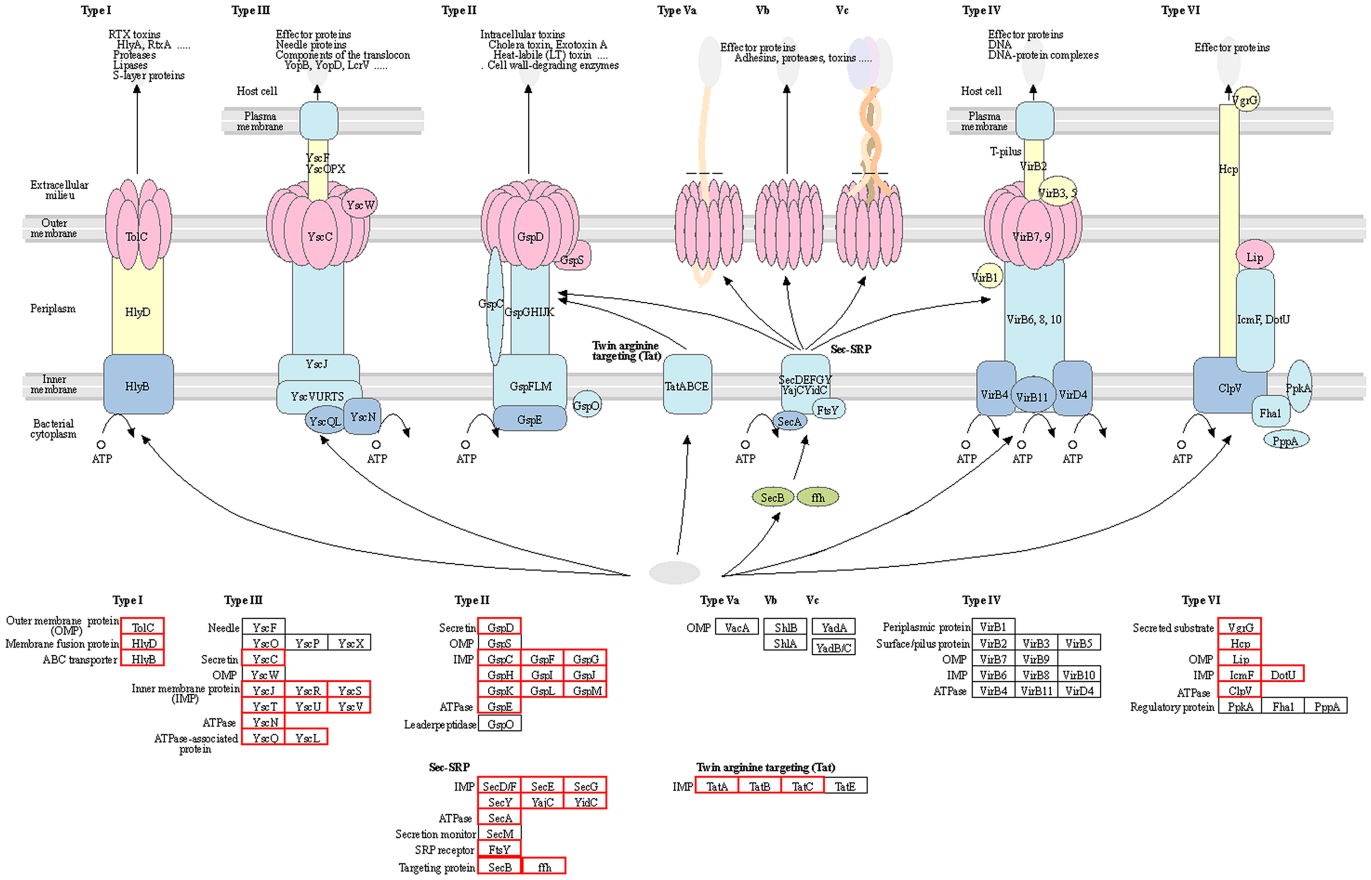
Statistical analysis of the structural composition of the secretion system of the gd-2 strain. The genes of each secretion system are represented by the gene names in the KEGG database, and the red boxes represent the corresponding genes in the strain.

To further investigate the pathogenic mechanism of *R. solanacearum* gd-2, we predicted the structural genes of its various secretion systems. Among them, 66 secretion system structural genes were identified, including five T1SS, 23 T2SS, 10 T3SS, 16 T6SS, 11 Sec-SRP, and three twin-arginine protein translocation (Tat). The statistical analysis of the structural composition of the secretion system strains is shown in Figure 5. Among them, the T1SS secretion system includes *tolerant colicin* (*tolC*), *hemolysin D* (*hlyD*) and *hlyB*. The T2SS secretion system includes *gspF*, *gspE*, *gspD*, *gspM*, *gspL*, *gspK*, *gspJ*, *gspI*, *gspH*, *gspG*, and *gspC*. The T3SS secretion system located on plasmidA *includes yersinia* (*ysc*) gene *yscL*, *yscJ*, *yscU*, *yscV*, *yscQ*, *yscR*, and *yscS*. The T6SS secretion system includes *valine glycine repeat protein G* (*vgrG*), *intracellular multiplication protein L* (*impL*), *hcp*, *vasD*, and *impK*. The secretion system and signal recognition particle secretion system (Sec-SRP) includes *secA*, *secB*, *secD*, *secE*, *secF*, *secG*, *secY*, *ffh*, *yajC*, *ftsY*, and *yidC*. The Tat secretion system includes *tatA*, *tatB*, and *tatC*.

A total of 179 genes of the two-component signal transduction system were identified in the genome of gd-2, including 101 regulator genes, 60 sensor genes, and 18 hybrid genes (Supplementary Table S13). In addition, a total of 34 chemotaxis-related genes were predicted in gd-2 (Supplementary Table S14). 99 quorum sensing-related genes were predicted (Supplementary Table S15).

### 3.5 Comparative analysis of T3E analysis for gd-2 and other *Ralstonia solanacearum* strains

**Figure 6:**
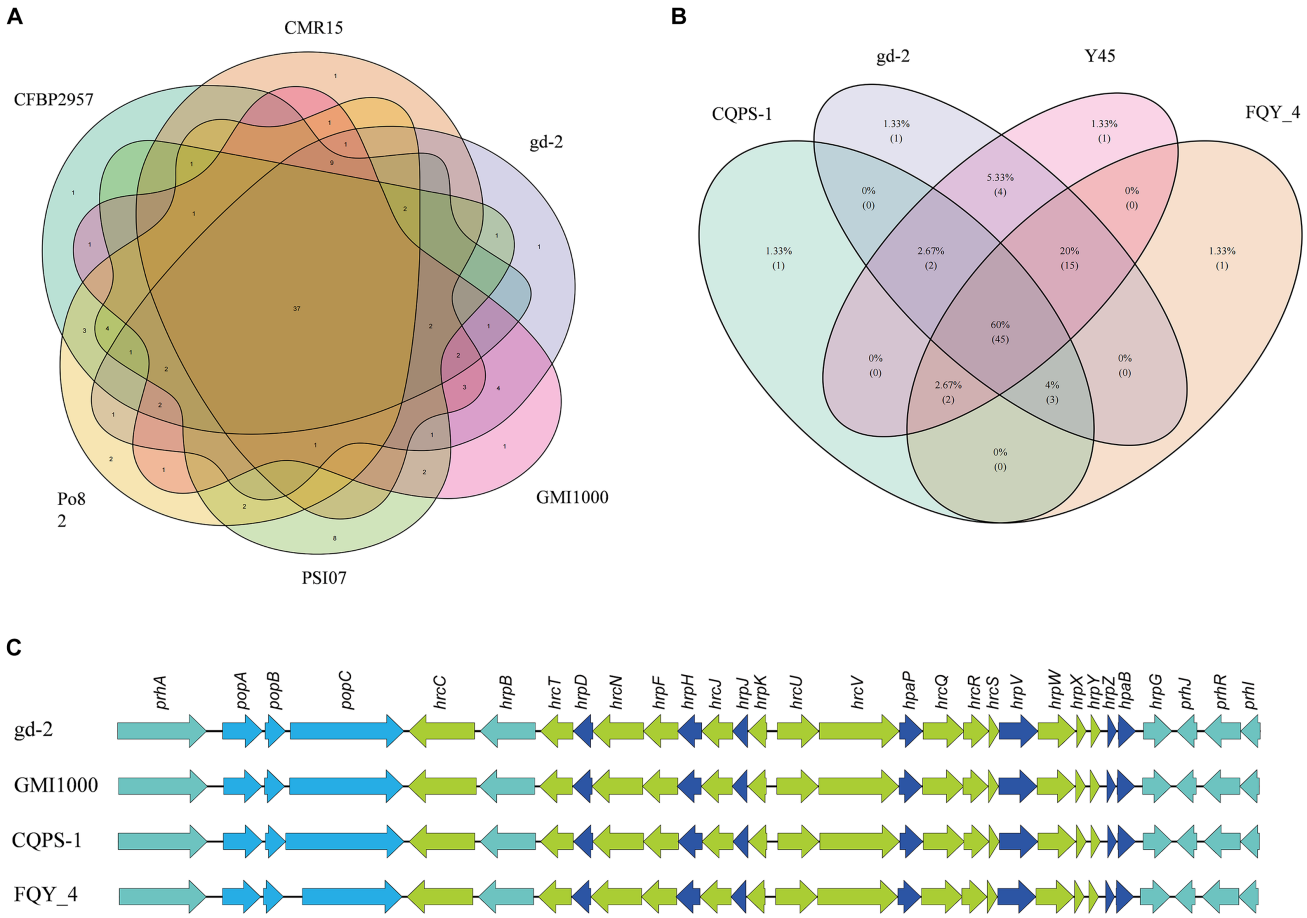
Venn diagram and gene comparison of *hrp* clusters among gd-2 and others *R. solanacearum* strain. **(A)** Venn diagram showing numbers of specific and shared gene families among gd-2 and five different phylotype species. **(B)** Venn diagram showing numbers of specific and shared gene families among gd-2 and three phylotype I species. **(C)** Genetic organization and gene comparison of *hrp* clusters between CQPS-1, Y45 and FQY_4.

The nine strains of *R. solanacearum* were identified to have 54–75 candidate T3Es, with CQPS-1 (54) having the fewest T3Es and Po82 (75) having the most. Total 72 T3Es were identified from strain gd-2, which was comparable to the number of T3Es in GMI1000 (74), FQY-4 (70), and Y45 (69), all of which belong to phylotype I. Comparing the T3Es of gd-2 with different strains of *R. solanacearum*, 37 T3Es were shared by six strains, and the T3E unique to gd-2 was RipAZ2 (Figure 6A). The T3Es unique to strain PSI07 were RipA, RipBB, RipBF, RipE1_1, RipE1_2, RipG1_2, RipG1_3 and RipH4; the T3Es unique to Po82 were RipA5_1 and RipA5_2; and the T3Es unique to CMR15, GMI1000, and CFBP2957 were RipG8, RipAH, and RipK, respectively. Among gd-2, CQPS-1, Y45, and FQY-4, 45 shared T3Es were identified (Figure 6B). Each of the four strains contained a unique T3E, with RipAZ2 for gd-2, RipP2 for FQY_4, RipT for Y45, and RipBE for CQPS-1. The candidate T3Es comparison betwwen gd-2 and other eight strains showed that 17 T3Es were shared, including RipA2, RipA4, RipA5, RipAB, RipAN, RipE1, RipF1_1, RipF1_2, RipG6, RipH3, RipI, RipN, RipS1, RipS3, RipS4, RipTPS, and RipW (Table 1). Most T3Es had high similarity among different strains, but there were also cases of deletion, indicating that both conserved and specific T3Es exist in different strains.

The functional genes related to the *hrp* gene cluster in all nine samples were statistically analyzed. The number of functional genes related to the *hrp* gene cluster identified in different strains ranged from 26 to 30, with 30 *hrp* genes identified in gd-2 (Supplementary Table S15). The *hrp* genes in gd-2 were consistent with the number found in GMI1000 and CQPS-1. A comparative display analysis of the *hrp* gene cluster was performed for 4 samples, including GIM1000, CQPS-1, FQY-4, and gd-2 (Figure 6C).

## 4 Discussion

The *R. solanacearum* species complex (RSSC) has complex species types. According to the sequence data of the 16S-23S rRNA gene spacer region (ITS), *hrp* and *egl* genes, RSSC can be divided into four phylotypes (Phylotype I, II, III, and IV). The reported phylotype I *R. solanacearum* includes sequence variants 13, 14, 15, 17, 34, 44, 54, 55, etc. (Zheng et al., 2014; Liu et al., 2017). In Chian, sequevars present distribution difference at different tobacco planting zones, sequevars 15 has a high percent at in various planting zones, such as nanling hilly area (46.15%), wuyi hilly area (46.15%), huanghua plain area (100%) and yimeng hilly area (100%) (Liu et al., 2019). The genomic data of Ralstonia isolated from tobacco have been published, including Y45 (sequevar 17), FQY-4 (sequevar 17), CQPS-1 (sequevar 17), FJ1003 (sequevar 14) (Cao et al., 2013; Liu et al., 2017). Thus, we processed comprehensive genome sequence analysis of sequevar 15 to provide new evidence for ultimately analyzing the pathogenic specificity of *R. solanacearum* and the prevention and control of bacterial wilt. Through amplification and sequencing, gd-2 was identified as phylotype I sequevar 15. According to comparative genomic analysis, gd-2 maintains relative consistency with other strains in both the chromosomal and plasmid genomes. This study is the first reported whole-genome sequencing study of sequevar 15, providing new data in support of exploring the regulatory mechanism of virulence differentiation and host adaptation in *R. solanacearum*.

The composition and diversity of *R. solanacearum* groups caused by bacterial wilt are very complex. Nowdays, 55 sequence variants of *R. solanacearum* have been identified and the NCBI database has published the complete draft genome of 145 *R. solanacearum* (Ahn et al., 2011; Cai et al., 2015; Liu et al., 2017; Asolkar and Ramesh, 2018; Greenrod et al., 2023). The genome of *R. solanacearum* is approximately 5.8 Mb, dominated by two circular replicons, with the occasional presence of small plasmids, such as CMR15 containing a 35 kb small plasmid and PSI07 containing a 13 kb small plasmid (Liu et al., 2017). The genome size of gd-2 was 5.93 Mb, including the chromosomes (3.83 Mb) and the megaplasmid (2.10 Mb), which was larger than phylotype I strain FJ1003 (5.90 Mb), phylotype I sequevar 17 strain CQPS-1 (5.89 Mb) and phylotype I GMI1000 (5.8 Mb) (Salanoubat et al., 2002; Liu et al., 2017; Chen et al., 2022). Gene prediction results showed that 3,434 and 1,640 genes were identified in the chromosomes and plasmids of gd-2, which were similar with FJ1003 (3,446 chromosomes genes and 1,564 megaplasmid genes), CQPS-1 (3,573 chromosomes genes and 1,656 megaplasmid genes), phylotype I sequevar 14 M strain FJ1 (3,502 chromosomes genes and 1,596 megaplasmid genes) (Tan et al., 2022). The *hrp* gene cluster is an important component of the T3SS, which is necessary for the pathogenicity of *R. solanacearum* and can induce hypersensitivity reactions in non host plants (Lindgren, 1997). And 30 *hrp* genes identified both instrain gd-2 and strain CQPS-1. However, the numbers of GIs which is important forms of horizontal transfer elements of gd-2 was less than CQPS-1 (21), FJ1 (21), and FJ1003 (23), which may affect the adaptability of bacterial strain gd-2 to the environment. The predicted noncoding RNAs of gd-2 included 59 tRNAs, 12 rRNAs, four 5S rRNAs, four 16S rRNAs, and four 23S rRNAs. The number of tRNAs is similiar to CQPS-1 (58), biovar 4 Bs715 (59), FQY-4 (62), and FJ1 (59), significantly higher than FJ1003 (35), phylotype I YC45 (46), and Race 4 Biovar 4 SD54 (46) (Cao et al., 2013; Shan et al., 2013; She et al., 2015; Liu et al., 2017; Chen et al., 2022; Tan et al., 2022; Jeong et al., 2023). And more rRNA may improve protein synthesis ability and improve the adaptability of strains to the environment.

Pathogenic bacteria rely heavily on effector molecules secreted extracellularly or directly into host target cells to induce toxicity in the host or surrounding organisms. These different functional macromolecules are transported extracellularly through different secretion apparatuses (Li et al., 2012; Bai et al., 2018). Currently, seven types of secretion systems have been identified, which exhibit diversity not only in the effector molecules secreted but also in the composition of the apparatus. T1SS, T2SS, T3SS, T5SS, and T6SS are mainly found in gram-negative bacteria, while T7SS is mainly found in gram-positive bacteria. T4SS is found in both gram-positive and gram-negative bacteria (Parizad et al., 2016; Cordsmeier et al., 2022). T1SS and T5SS contain simple structure, consisting of only two or three proteins (Zhou et al., 2019). T2SS, T3SS, T4SS, and T6SS exist more complex than T1SS and T5SS and their apparatus can traverse the entire cell membrane (Korotkov et al., 2012). Study on T7SS is still in its infancy, and the specific apparatus and mechanism are still unclear (Bitter et al., 2009). T3SS, T4SS, and T6SS can directly inject effector molecules into eukaryotic cells, and they are mostly encoded by clusters of consecutive genes, especially in pathogenic bacteria, where these apparatus genes often exist as virulence islands (Liao et al., 2021). In this study, 66 secretion system structural genes were identified, including 5 in T1SS, 23 in T2SS, 10 in T3SS, 16 in T6SS, 11 in Sec-SRP, and 3 in Tat.

The T3SS effector proteins have an *hrp* II-box (TTCGN16-TTCG), which is activated by *HrpB* and *HrpG* transcription and powered by the ATPase complex. It enters plant cells through the cytoplasmic ring, basement, endomembrane exit, and transport pore (Mcnally et al., 2011). The effector proteins in *R. solanacearum* consist of 94 orthologous families, of which 71 are transferred or secreted through T3SS (Cunnac et al., 2004). A total of 72 T3SS proteins were identified in the strain gd-2 genome and 72 T3Es were identified, which is comparable to the number found in GMI1000 (74), FQY-4 (70), and Y45 (69). RipAZ2 is a unique T3E in gd-2 compared with other eight sequenced strain. However, there are significant differences between CQPS-1 (54) isolated from phylotype I of tobacco and CMR15 (61) isolated from phylotype III. The framework division of *R. solanacearum* lineages result from the evolution and geographical origin of *R. solanacearum*, so it is speculated that the T3Es specific to each of the four *R. solanacearum* lineages may have been formed during the long-term evolution of the strains and their hosts, which also reflects the complexity of *R. solanacearum* species from another perspective.

## Data availability statement

The data presented in the study are deposited in the NCBI repository, accession numbers PRJNA1071833 (BioProject) and SAMN39714303 (BioSample).

## Author contributions

ZX: Methodology, Resources, Software, Validation, Visualization, Writing – original draft. GL: Formal analysis, Funding acquisition, Investigation, Writing – review & editing. AY: Formal analysis, Funding acquisition, Investigation, Writing – review & editing. ZL: Software, Visualization, Writing – review & editing. MR: Software, Visualization, Writing – review & editing. LC: Software, Visualization, Writing – review & editing. DL: Software, Visualization, Writing – review & editing. CJ: Conceptualization, Writing – review & editing. LW: Conceptualization, Writing – review & editing. SW: Conceptualization, Writing – review & editing. YC: Conceptualization, Writing – review & editing. WY: Resources, Writing – review & editing. RG: Resources, Writing – review & editing.

## Footnotes

^1^ https://www.ncbi.nlm.nih.gov/genome/genomes/490

^2^ https://iant.toulouse.inra.fr/bacteria/annotation/site/prj/T3Ev3/

^3^ http://sequence.toulouse.inra.fr/R.solanacearum

^4^ http://www.pacb.com

^5^ https://github.com/marbl/canu

^6^ https://github.com/broadinstitute/pilon

^7^ http://ccb.jhu.edu/software/glimmer/index.shtml

^8^ http://trna.ucsc.edu/software/

^9^ https://github.com/tseemann/barrnap/

^10^ https://web.expasy.org/docs/swiss-prot_guideline.html

^11^ http://pfam.xfam.org/

^12^ https://geneontology.org/

^13^ https://www.kegg.jp/kegg/

^14^ http://github.com/bbuchfink/diamond

^15^ http://www.circos.ca

^16^ https://www.pathogenomics.sfu.ca/islandviewer/

^17^ http://phaster.ca/

^18^ http://www.mgc.ac.cn/VFs/main.htm

^19^ http://arpcard.Mcmaster.ca

^20^ http://www.cbs.dtu.dk/services/TMHMM-2.0/

^21^ http://www.cbs.dtu.dk/services/SignalP

^22^ http://sourceforge.net/projects/brig/

^23^ http://mjsull.github.io/Easyfig/
